# Psychoanalytic Underpinnings of Socially-Shared Normativity

**DOI:** 10.3389/fpsyg.2019.02032

**Published:** 2019-08-30

**Authors:** Michael Forrester

**Affiliations:** School of Psychology, University of Kent, Canterbury, United Kingdom

**Keywords:** psychoanalytic, theory, ethnomethodology, social, sharing

## Abstract

Alongside social anthropology and discursive psychology, conversation analysis has highlighted numerous ways in which cultural forms of perceiving and acting in the world are primarily rooted in socially shared normativity. However, when consideration turns to the origins and purposes of human affect and emotion, ethnomethodology, and conversation analysis appear to face particular difficulties that arise from the over-arching focus on sense-making practices. This article considers the proposal that psychoanalytic thinking might inform our understanding of how socially shared normativity emerges during infancy and early childhood. First, a framework is sketched out that highlights the fact that from the beginning, an infant’s earliest experience is bound up with those procedures, practices, and social actions that make up what conversation analysts call members’ methods. Second, comparisons are drawn between conversation analysis and psychoanalytic accounts of early experience for infants during the first years of life. Discussion then moves to the Kleinian notion of object relations and the concept of projective identification. Essentially, this is a theoretical account of how “what-was-once-one” (the mother-infant unit) somehow differentiates resulting in the gradual emergence of the “individuated being.” What is often glossed over in this account is the discursively embedded nature of projective identification; a process that is itself interdependent with the embodiment that makes up the infant’s lived engagement with the world. Whatever might constitute consciousness emerges from somatic, embodied, material-physical, tactile/affective experience – that is, a fundamentally social milieu. Ultimately, this raises the question of how transformation (i.e., from the social to the individual) occurs. One answer may be Winnicott’s idea of the transitional space, where the “good-enough” parent is said to be somebody, who can “contain” both negative and positive identifications coming from the infant, transform and re-project such identifications, but in modified form. In this way, the infant begins to recognize/experience what it is they are “feeling.” Such projective identifications are conveyed within and through the prevailing discourses that constitute all social practices. Concluding comments note that conversation analysis may find in psychoanalytic thinking a framework for understanding the interdependence between affect and action, given that in psychoanalytic thought, we find a thoroughly relational conception of human nature.

## Introduction

Over at least last 50 years or so, since Sack’s (1992) seminal lectures, there is little doubt that alongside social anthropology and discursive psychology, conversation analysis has highlighted numerous ways in which cultural forms of perceiving and acting in the world are rooted in socially shared normativity. However, when consideration turns to the origins and purposes of human affect and emotion, conversation analysis appears to face particular challenges regarding the relations between action and emotion that may arise from the over-arching focus on sense-making practices found in this approach (Sorjonen and Peräkylä, 2012). As follows, the suggestion will be made that the researchers interested in the primary roots of socially shared normativity may have an unrecognized difficulty with breaking away from deeply held assumptions regarding affective or emotional dimensions of human experience, particularly the notion that these remain private or individuated (pertaining only to a specific individual’s private experience). In order to help dispel or disabuse such unrecognized presuppositions, a case is made for considering new psychoanalytic approaches to affect and emotion, where one finds a socially saturated conception of what constitutes psychological life – specifically in what have become known as object-relation approaches.

The first part of the article will highlight research in conversation analysis (CA form here) that has explicated the fact that from birth, an infant’s earliest experience is bound up with those procedures, practices, and social actions that make up what are termed members’ methods. Observations will then be offered that draw attention to a certain avoidance or elision regarding the domain of affect and to detailed discussion of what kind of implicit model(s) of subject-hood are oriented to by researchers in child-focused CA. Having highlighted challenges CA work faces when studying the interdependence of affect and action, the second part of the article outlines a partial summary of developmental psychoanalytic thought, specifically some of the key ideas in the object-relations school. This forms the basis for suggesting that child-focused CA may find this perspective a rich source for developing a discourse of affective-normativity.

## Child-Focused Ethnomethodologically Informed Conversation Analysis

From the beginning, an infant’s earliest experience is bound up with the practices and social actions that make up what ethnomethodology call members’ methods. In the emerging child-focused conversation analytic literature, there are indications that the domain of what constitutes “talk-in-interaction” has expanded considerably. Numerous studies document and explicate the diverse range of social practices that together form the rich nexus of multi-modal events that together form the acculturation frame for the infant and young child. We find representative examples of recent work for instance on touch, embodiment, crying, laughter, whining, pleasure, and affect (Wiggins, 2002; Laurier and Wiggins, 2011; Fantasia et al., 2014, 2015a,b; Cekaite, 2015; Jenkins and Hepburn, 2015; Berducci, 2016; Walker, 2017; Butler and Edwards, 2018). This research seems to indicate that in child-focused conversation analysis (CA from here), the boundaries of what would normally come under the umbrella term “talk-in-interaction” continue to expand. However, there may be a slight ambiguity or unrecognized difficulty with such expansion as it seems to indicate a move away from the methodological foundation stone (or lodestone) of reflexive accountability. Consider the following comment from Livingston (1987) that highlights the all-pervasive nature of the ethnomethodologically informed CA project:

What the common person knows or does not know is not at issue. Instead, the central issue and the central research problem is the examination of the unwitting, without extrinsic motivation, production of the ordinary social object. …[it is a] massive domain of phenomena - the domain of practical action and practical reasoning. It is this omnipresent domain of **practical methods**, *through which* and wherein people **make of the things they are doing** the **things that they accountably are**, that the ethnomethodologist seeks to investigate. By examining those methods in the material detail of their always-idiosyncratic embodiments, the ethnomethodologist seeks to understand those methods in and as that same, endlessly diversified, identifying specificity. (p. 12 – emphasis in original)

In effect, *all* social practices are open to analysis, including the practices and procedures of scientists, social researchers, and conversation analysts themselves. A couple of things are noticeable about this programmatic statement. First, there is the elision or avoidance of terms and concepts that presuppose knowledge, mental states (and one would surmise, emotion and affect), and anything that might be said to be “inside” or private to the individual. While this reflects the healthy skepticism CA and discursive psychology exhibit toward the logocentric excesses of traditional psychology (Coulter, 1999; Edwards and Potter, 2005), we can ask whether such elision may raise difficulties for understanding affective dimensions of talk-in-interaction. As Sorjonen and Peräkylä (2012) put it, “we do not yet have a satisfactory understanding of the relation between action and emotion.” (p. 9).

Second, methodic practice is both omnipresent and at the same time, evidenced through procedures of reflexive accountability, a competency that an infant or young child is unlikely to possess and which thus positions her in a kind of ethnomethodological limbo. The classic CA position regarding membership of a culture is that it is something that is gradually attained, in the sense that a child has to learn those performances and practices that constitute “doing” membership appropriately (Garfinkel and Sacks, 1970). However, as Shakespeare (1998) argues, because children are not effectively full members, the child’s role in interaction is constructed in terms of them building toward becoming a competent person, where much of their experience is replete with examples from adults concerning how to achieve full membership. Do infants and young children learn how to display affect and emotion from adults – simply as a set of actions or stances? Or do we assume that (spontaneous, innate) emotion states are molded by adults such that display reflects cultural dimensions of normativity? Questions of this nature seem to indicate a role for developing a discourse of affective-normativity.

Becoming a full member then presupposes possessing the skills and competencies that surround whatever it is taken as the appropriate performance or display of emotion. Sorjonen and Peräkylä (2012) suggest that perhaps the closest relation between emotion and action (methodic practice) is to be found in “displays of emotion that, at least in some context, can be considered as action” (p. 9). If we think about the areas where expressions of affect are being studied (such as crying, whining, complaining, and laughing), it seems that child focused CA is moving closer to a position where affect and emotion is conceptually associated with normativity. Whatever we understand by the terms affect and emotion, the question remains whether this is something – a dimension or domain – that remains unique (private) to the individual, or instead part of those members methods (social practices) that ultimately depend on learning what counts as appropriate performance.

Some discussion regarding normative dimensions or domains of affect seems important given the emergence of infant and child-focused CA work. Recent research documenting and describing the subtle and delicate nature of multi-modal social practices underpinning early parent-child talk-in-interaction is drawing attention to the social-semiotic embodied dimensions of affective-normativity (e.g., Cekaite, 2016; Kern, 2018; Holm Kvist, 2018). From the outset, whatever we are calling the infant’s “experience” or consciousness is interdependent with the specific social and cultural practices surrounding birth, childhood, dependency, mothering, asymmetry – i.e., whatever makes up the matrix of social practices surrounding the infant. However, even to use a term such as “surrounding” immediately draws attention to difficulties regarding what is presupposed by categories and constructs such as personhood, individuality, separation, subject, and object, and in fact, all those terms and associations brought into play when we seek to understand what makes for a socially shared normativity. One can imagine that a CA perspective on what an affect-focused socially shared normativity would constitute, would be one where whatever we understand as mutual engagement, is something where the mutuality being displayed is viewed as evidence that there exists (and is in play) a normative system oriented to by the participants who are involved in “doing engagement” or joint participation. However, can one successfully “perform” doing-being-emotionally engaged, such that said performance is open to scrutiny and reflexive accountability? Part of the difficulty surrounding thinking about affective dimensions of normativity (i.e., those conventions and practices associated with the display and performance of emotion) may be linked to the empirical requirements of CA, particularly that analytic interpretation should rest upon identifiable participant-oriented evidence in talk-in-interaction itself. Such a requirement may engender an avoidance for developing a theoretically informed discourse or set of descriptions for what constitutes affect or emotion.

Recent work in CA highlights the various interdependencies between sequential organization and the display of emotion or participant’s “emotional stance” (Goodwin, 2007; Stivers, 2008; Voutilainen et al., 2014), and understandably this line of work exhibits a pervasive focus on the performance details of the fine-grained orderliness, in what one might call an example of methodological “affect avoidance.” Maynard and Freese (2012), for example, in a subtle and detailed analysis, draw out the significance of intonation during the on-going production and reception of good and bad news, making the point that their approach,

Shares the constructionist commitment to studying display of emotion in interaction and *remaining agnostic about the existence* of internal accompaniments to such displays. (p. 94). [emphasis added]

What is interesting here is that this agnosticism nevertheless presupposes a possible backdrop of internal emotional states – which remains beyond discussion (for empirical reasons). Similarly, for Goodwin and Goodwin (2000) emotion is a social phenomenon that is made visible or constructed in and through the systemic practices lodged within the processes of situated action, “used by participants to build in concert with each other the events that make up their life world” (p. 569). What does warrant comment is the difficulty that CA and related discursive approaches appear to have regarding terms such as affect and emotion.

Building on work in philosophy, aesthetics and critical theory (e.g., Massumi, 2002; Deleuze and Guattari, 2013) a number of writers emphasize a recent *affective turn*, characterized as a movement toward understanding domains of experience outside of the dominant paradigm of representation (Clough and Halley, 2007). In a recent special issue on affect and subjectivity, Lara et al. (2017) make the point that the “missing subject” is one of the predominant critiques of the turn to affect, where there is “considerable unease about what a vacated subject meant for questions of power and agency.” (p. 32).

Examining these developments, Wetherell (2013) describes the aim of affect theory [an approach that emphasize processes, *beyond*, *below,* and *past* discourse, (e.g., Massumi, 2002; Thrift, 2008)], as a perspective that aims to “deliver the tools required for lively, textured research on embodied social action and for productive insights into the entangled forms of assembling constituting social life moment to moment.” (p. 351). Describing example views of affect theory, Wetherell (2013) comments on Massumi’s (2002) *affect as excess* viewpoint, where:

“He [Massumi] maintains that affect is thus a kind of intensity, making a difference below the threshold of consciousness, thrusting the subject into particular kinds of relations with the material, and social world…[and]…discourse works on a different track from affect – a ‘quality’ track as opposed to the ‘intensity’ track. The quality track leads to naming, and to the framing of affect in conventional discursive, linguistic and cultural terms. If affect is a kind of chaotic excess and the unprocessed push, then the moment of discursive representation is bureaucratic and organizational. For Massumi, it is the process by which potentially ‘wild’ affect is tamed, turned into something people can recognize, talk about to each other and communicate as ‘domesticated’ emotion.” (p. 354).

Attempting to build a productive dialogue between traditions in discourse studies (e.g., CA) and new lines of research in affect and emotion, Wetherell (2013) examines a sequence from Goodwin’s (2006) work on children’s playground games, noting that Goodwin seeks to explore affect and discourse equally, assuming that, “these are entangled in the sense that embodied action (on a scale of intensity) tends to be bound up with talk at some point in a flow of activity” (p. 360). Her analysis concludes that research such as Goodwin’s (2006) moves beyond a simple binary divide of “affect vs. discourse” given that this work,

effectively conveys the feel and patterning of bodies in action, the lively flow of social life and sticks closely to participants’ perspectives…(and)…it puts both affect and discourse back where they should be within emergent patterns of situated activity, and makes the patterns, as they need to be, the main research focus. (Wetherell, 2013, p. 364).

Certainly, there is little doubt that the Goodwin’s have been significant in developing the notion of affective stance loosely defined as “a positioning accomplished through conduct and thereby made publicly accessible” (Sorjonen and Peräkylä, 2012, p. 5). Whatever affects are within CA, they can be utilized as resources – something that people in talk-in-interaction can draw on. Following their helpful explication of bodily compliance by children Goodwin et al. (2012) argue that alongside the traditional study of facial expression and the psychology of emotion, research should consider,

the relevant actions and bodily displays of the parties they are interacting with. We argue specifically that the body of the party producing an emotional display cannot be examined in isolation. Crucial to the organization of emotion as public practice is the way in which individuals display rapidly changing stances toward both other participants, and the actions currently in progress. (p. 39–40)

For Goodwin et al. (2012), affective stance and emotion are not “add-ons” but “constitute central components of the situated actions participants build to carry out the mundane activities that make up the lived social world they inhabit together.” (p. 40).

Interestingly, such comments are not so far removed from those emotion theorists in developmental psychology who 20 years ago, and coming from the opposite direction, called for a move away from a focus on the unifying role of a “central feeling state” toward a realization of what the child is doing to adapt his or her goals to the environment, and to modify the environment to fits said goals (Campos et al., 1989). Described as “emotion regulation,” displays of affect involved, “regulating the action tendencies of the other facilitating action tendencies when desirable, redirecting them when necessary, or preventing them when culture or danger dictates” (Campos et al., 1989, p. 397). While understanding, what does seem clear is that in CA theoretical elaboration regarding discourses of affect and emotion tends either to be avoided or deemed unnecessary, given that whatever it might be, it is reducible to social praxis – will always remain an empirical question linked to requirements of participant-orientation and the display of methodic practice. This suggestion here is that such a constraint may be unhelpful when considerations turn to theoretical underpinnings of socially shared normativity.

Before moving to the main focus of this article, certain psychoanalytic perspectives on affect and emotion (with regard to discourse, methodic practice, and normativity), some comments are warranted regarding conceptions of the infant/child’s mind we find in CA. Understandably, given the work that CA and discursive psychology have done so as to provide and alternative view to that found in traditional developmental psychology (Leslie, 1987; Perner, 1992) commentators are certainly suspicious or circumspect about presupposing a foundational or causal significance to “internal” development (e.g., cognitive development, emotional maturation, neurobiological change and so on). In their critique of the overemphasis on “theory of mind” and its relation to cognitive competence in developmental psychology, Lerner et al. (2011) make the point that cognitive representational conceptions of underlying skills should conform to, “the actual requirements of the observable interaction order and participant in it – for example, the structurally afforded ability to recognize, project, and contingently employ unfolding structures of action in interaction with others.” (p. 45). For CA whatever cognitive capacities are found to underwrite interactional order, the specification of the relevant elements of this domain requires a close and systematic analysis of naturally occurring interaction addressed to the manifold contingencies of everyday life, and the social-sequential structures that enable human interaction. Lerner et al. (2011) comment;

It seems to us that very young children only require the *in situ practiced capacities required* to recognize, in each particular case, the formal structures of the in-progress actions that recurrently fill their social interactional world and the practical skills to participate in each context-specific realizations of those structures of action as they are progressively realized, and as each next element in its progressive realization, projects a next constituent of that structure. (p. 57).

What underscores CA child-focused analysis, is that while there may be some recognition that social interaction may in part depend on evolved neural mechanisms of (an individual’s) brain, there is no defensible basis for the presupposition that the skills and competencies employed derive from cognitive representational entities in the mind. For Lerner et al. (2011) the young child’s abilities are to be understood as akin to affordance-like capacities intimately connected with detecting patterns in the ongoing sequence of actions and events made available to them through talk-in-interaction.

Similarly, in recent work by Keel (2015), when summarizing pre-school children’s skills and competencies when building up a normative position of the surrounding world, comments;

my detailed study of how children deploy assessments to achieve self-praise, noticings, announcements, complaints, or requests displays their orientation toward participants’ membership categories, the responsibilities and rights that are bound to them, and the larger praxeological context, adapting their way of packaging their initial assessment and mobilizing different sequential, formal, linguistic and embodied resources accordingly. (p. 218).

The picture of the child’s mind here is of an entity who can “package assessments” and adapt them to circumstances, and mobilize resources of various kinds. The entity is certainly constructivist but further commentary on what constitutes the “being-who-is-constructing” is avoided or evaded. A similarly cautious or circumspect perspective can be seen in the earlier work of Wootton (1997) when discussing children’s emerging competences underpinning their capacity to use local and public understanding(s):

Around that time (aged two years old) the child develops the skill to identify and draw on local knowledge which has been made apparent within prior interaction. Because this knowledge is contingent and local I have chosen to use the term “understanding” to describe it rather than a term like “representation,” the latter indexing forms of knowledge which have a more enduring status within the mind. (Wootton, 1997, 192–193).

Notice that understandings are something that are now public and accountable – social objects produced and reproduced in the ongoing dynamics of interaction. However, the idea or notion of the child’s mind (as nevertheless existing and being something separate from that which is experience) remains in the background in CA. Beyond the assumptions that this agent is a learning being who accrues the skills and abilities to employ available resources (e.g., the competencies *to draw on local knowledge*), discussion regarding emotion or affect seems to be something to be avoided.

There are then a number of challenges that CA faces when seeking to understand the relationship between action and affect/emotion, particularly for child-focused research. The members method criteria underpinning membership status (e.g., reflexive accountability) seems to be glossed over once the detail of adult-child engagement and participation begins to be examined (Forrester and Reason, 2006; Filipi, 2009). In addition, while considered and important insights have come from research on affective stance, such insights still seem to rest on the possible existence of person-experience affect/emotion state(s). There remains an understandable reluctance to discuss or develop a conceptual framework or discourse regarding affect. Parallels to such avoidance or elision can be found in the guardedness or skepticism regarding cognitive dimensions of the developing child in early child-focused CA work. Although such caution has helped counter the excessive formalisms of the dominant and traditional approaches in disciplines such as developmental psychology (e.g., Leslie, 1987; Perner, 1992); this seems to have left a vacuum or absence when it comes to trying to think through what a discourse of affective-normativity might look like. Psychoanalytic approaches may offer some helpful suggestions in this regard, as other critical theorists have pointed out (Frosch, 2003; Hollway, 2011).

## Object-Relations Perspectives on Affect

We can begin by noting that the psychoanalytic developmental account of the emergence of emotion or affect comes from a perspective that is not only at odds with CA accounts but is somewhat different from the dominant views found in developmental psychology. In psychoanalytic thinking, the forces at play in the mind are dynamic and unceasing and motivated by primitive and ultimately biologically oriented forces of energy, both positive and negative (traditionally termed “instincts”). The objects and entities said to make up the unconscious are a motley collection of undesirable, and unrealizable/incomprehensible elements, some constitutional others acquired and constantly seeking to undermine whatever we understand as the coherence of the ego. This is a view of mind where the human (adult, child, or infant) is a being who possesses certain attributes and characteristics of mind that forever seek to undercut whatever notions one has of possessing a stable mind (conscious-self) entity. Leaving aside the long-discussed issues surrounding methodology and empirical support^1^, this perspective certainly stands in stark contrast to the perspective found in CA or in contemporary developmental psychology.

One particularly different and noticeable aspect about the psychoanalytic view of the developing child is the idea that from the beginning the issue of separateness and “self-identity” is called into question – this is the significance of the Freudian legacy of the later 19th century. Rather than just assuming there is a sense of separateness accompanying the infant’s experience of the earliest moments of life, the psychoanalytic view asks under what conditions are we to understand how an infant “attains” or moves to the position of experiencing “separateness” or “individuation” in the first place? Psychoanalytic thought requires or demands a critical examination of any assumptions and presuppositions surrounding awareness, self, or whatever we might want to call consciousness of separateness. Theoretically developed accounts of the developing self are to be found in the object-relations view of Klein (1949, 1957) and Winnicott (1960, 1974), and in recent psychosocial approaches (e.g., Stern, 1985; Hollway, 2006; Walkerdine, 2014).

The psychoanalyst Donald Winnicott, for example, consistently emphasized the role of affective states said to be (constituted) by movement between states of ego disintegration and partial integration of “self-awareness.” In this account to “exist” as an infant at all is to some extent an achievement. To paraphrase Green (1999), Winnicott’s unique contribution was to show that at the beginning the notion of a separate baby is incoherent, and that,

it is necessary to include the mother in the indissoluble couple that they form. *That is to say, no discourse on the affect can be sustained that does not take account of the mother’s affects*, her tolerance of the child’s regressive needs, even of a state of informal chaos, the necessary conditions for the establishment of a kernel of vital affective continuity (emphasis in the original, p. 77).

Psychoanalysts such as Green (1999) draw attention to the constraining representation-affect opposition prevalent in psychology and philosophy and instead seek to confront the affective basis of the sense of existence, as well as encouraging the development of an affect discourse. Possibly one of the difficulties we encounter when addressing affect and emotion is the pervasive or all-encompassing (and yet elusive) nature of what it is we are trying to get at, or to quote Green (1999) again,

that the essence of affect is its dynamic attribute, its capacity to seep into other domains and inhabit them and finally to transform both itself and products of the area of the mind that it has occupied. (p. 285).

Part of the problem when thinking through what might constitute an affective-normative dimension to social life is the possibility that such a dimension undercuts or rather permeates all aspects of life. For now, let us continue the psychoanalytic narrative regarding beginnings and the emergence of early psychic life for the infant.

Building on Freud’s concept of instinct, the “object” of object-relations theory is anything that is employed by the instinct(s) in order to achieve its aim, Klein (1963) describes in detail how the child moves through different stages of psychic development, starting from an initial biologically determined state where both “life” and “death” instincts are in play. From the beginning the dangers, challenges, and opportunities engendered by these contrasting instinctual forces leads to a psychic splitting or differentiation of “good” and “bad” objects:

Even the child who has a loving relation with his mother has also unconsciously a terror of being devoured, torn up, and destroyed by her. (Klein, 1963, p. 277)

The model of the early mind is of a fragile ego that is sensitive to processes of dissociation and fragmentation – fragmentation due of the piecemeal way in which the world is “introjected,” and dissociative because there is the ever-present inherent (internal) danger expressed as anxiety (i.e., determined by the death instinct). Working on the assumption that the human organism is likely to come into the world with a rudimentary ability to sense danger, Klein associated life’s first experiences of anxiety not with acquired or learnt mental abilities, but with an internal registering of unconscious tendencies that Freud had termed the death instinct. In other words, survival meant that the baby was born knowing about death and sensing her internal destructive instincts, and this first knowledge took the form of a primordial terror or annihilation. Anxiety is thus basic to all living states, however immature, and it is this underpinning sense of danger and potential disintegration that gives rise to a spontaneous splitting. At this point is should be emphasized that this discussion focused on the earliest moments of infancy and young childhood – approximately the first year of life (leaving aside later the complications and challenges of sibling and peer socialization).

In a related commentary on the intersubjective approach to the self that originated in object relations theory, Hollway (2006) highlights the dynamic dimension of the unconscious, noting,

At a time before the infant can experience any self boundaries, these are provided by the mother…[and]… Bion (1967) saw this in terms of the container (mother) and contained (infant). Projective identification for him is a form on unconscious communication which enables a receptive mother to experience the feelings of her baby, transform them by using her mind, and through her body and emotional state communicate these modified, hopefully detoxified, feelings back to the infant, who can feel them to be bearable. The infant in this way borrows the mother’s mind, which only gradually becomes internalized to the point where it is the baby’s own resource. (p. 475).

Building on Klein’s ideas, Winnicott (1974) coined the well-known phrase the “good-enough” mother, said to be somebody who can “contain” both negative and positive identifications coming from the infant, transform and re-project such identifications, but which are now in modified from. The point worth emphasizing here is that such maternal or paternal projective identifications should be understood as part and parcel of ongoing unconscious relational dynamics. Hollway (2006) commenting on what relational or intersubjective psychoanalytic accounts have in common,

is the notion of a dynamic unconscious: “the way in which our mind transforms new relations into old ones (transference); others into parts of ourselves (introjection); and parts of ourselves into others (projection)” (Alford, 2002, p. 3), and it is this that distinguishes them from relational theories which revert to an idea of relationship between conscious, intentional bounded individuals. (p. 475).

One of the first puzzling questions we can ask is how the an extremely fragile ego constantly under threat of disintegration could *introject* and *project* in the first place, especially as these are said to be psychic processes that require some degree of stability and boundedness. Essentially what seems to be involved in holding even the first elements of what might constitute a personality or ego together, is that this “keeping together” experience is “performed initially from outside.” Bick (1968) suggests that the baby has to struggle for the capacity to introject, and that this achievement of both infant and mother is related to embodiment, “The stage of primal splitting and idealization of self and object can now be seen to rest on this earlier process of containment of self and object by their respective ‘skins’” (Bick, 1968, p. 484). Embodiment presupposes containment and the establishment of boundaries, and it would seem that before the infant can do anything at all, it has to experience an object in such a way that it intuits the concept of a space that can hold things. In other words, interdependent with the experience of being fed, is the creation of an “inside,” and that,

(Bick)… showed the baby struggling for the capacity to introject and that this is a function of the skin, or rather a function of skin sensations which arouse fantasies of a containing object …(and)…the first introjection is the introjection of an object which provides a space into which objects can be introjected. Before projection can happen there has to be an internal object capable of containing which can be projected into an object before that object can be felt to contain a projection. (Hinshelwood, 1989, pp. 193–4).

It is in this way that the creation of a unified space comes about, where before there was none. Only with the existence of an internal psychologically enclosing space can the capacity to introject emerge. The first achievement is to win the concept of a space that holds things, “the infant in gaining the nipple in his mouth has an experience of acquiring such an object – an object that closes the hole (the mouth and other orifices) in the skin boundary. (Hinshelwood, 1989, p. 194).” This experience is fundamentally rooted in the somatic-affective domain. Whatever might constitute consciousness emerges from somatic, embodied, material-physical, tactile/affective experience – that is, *a fundamentally social milieu*.

Although the Kleinian account of psychic development starts from neurobiological assumptions regarding survival and existence (e.g., instincts) the boundaries between what constitutes the “external” and “internal” are initially very blurred. In other words, while it is assumed that at some basic level the infant orients to the fact that the breast-sustenance (part-object) is very much external, it is just as much a construction from within, or as Kristeva (2001) puts it:

(an) internal image, to the extent that the fragile ego, as it constructs and deconstructs the boundary between the inside and outside, is where this quasi-object (or this object-being-constituted) is formed. From the outset, then, the *primal object* of the paranoid-schizoid position emerges, in Klein’s view, if and only if it is an *internal object* constructed through a fantasy of omnipotence.

The initial experience for the infant became known as the paranoid-schizoid position, so called in that it amounts to the totality of the infant’s instinctual desires and unconscious phantasies – where the libidinally invested breast as the primary good object reflects the power of the life instinct. For the infant the experience of the immediate satiation of hunger/distress is not something that is “happening-to-me” as a separate individual but rather a state of vacillating “omnipotence-to-pain/annihilation” (thus the term “paranoid-shizoid” position)^2^. The metaphor of positions and movement between and within them should be understood as a shifting affective-dynamic psychic vantage point. Gradually however, and realized in part through neurobiological maturation (around 3–6 months), the rudimentary and fragile elements/part-objects begin a gradual synthesis or coming together. There are both negative and positive aspects of this moving toward the rather sombrely termed “depressive position^3^”. The account here is that the infant begins to recognize that this gradually solidify “whole mother” is “understood to be the sole site of both sustenance and privation, and while this is much closer to reality, it necessarily ushers in a sense of the painful imperfections and limitations of life.” (Likierman, 2001, p. 101). A representative account is that:

The infant loses the precious sense that there exists, somewhere, an ideal object of unlimited pleasure and satisfaction. This triggers an experience of a “loss of the loved object.” The whole mother initially represents a despoiled perfection and provokes sorrow and indignant rage in turn. … It is this recognition that triggers the depressive position (Likierman, 2001, p. 101).

The depressive position, amongst other things, describes the initial recognition of awareness of separateness. In the paranoid-schizoid position there is no such awareness. Before such inklings there is in effect, no infant, in other words, no subjectivity, no experience, no memory. However, there is a history that is marked on the body – it is just that there is no word-based history. This is what Winnicott (1974) meant when he states there is *no such thing as an infant* initially, only the mother-infant unit. The danger or challenge of becoming human is that of relating to people who ultimately you have no control over (i.e., unlike in the paranoid-schizoid position with the phantasy of omnipotent control). Winnicott describes the infant as becoming capable of the capacity for “ruth” – the possibility of feeling concern for another person. This arises through the gradual awareness that another person is a subject as well as an object. Gaining relatedness and a sense of subjectivity involves the giving up and loss of omnipotence and unity-of-twoness (i.e., there is no awareness of separateness in the mother-infant unit). The assumption is that at some level this is an affective/emotional loss but nevertheless a necessary and required element of psychological development and growth. Hollway (2006) makes the point that Winnicott, and psychoanalysis more generally:

sees separation and the ability to differentiate between one’s own wishes and those emanating from outside as being crucial in the gradual achievement of self. Babies struggle to achieve unit status, and total independence is not the outcome of development. Winnicott (1968) understands children as proceeding from “absolute dependence, rapidly changing to relative dependence, and always travelling towards (but never reaching) independence” (p. 90). (Hollway, 2006, p. 476).

The gradual shifting away from the paranoid-schizoid position also engenders in the infant the desire to make reparation for the damage or destruction of the lost object (the phantasised internal object she used to have control over) who no longer exists. Hinshelwood (1989) comments that reparation, though it is concerned primarily with the state of the internal world and the good object (said to form the core of the personality) is usually expressed in action toward the mother. Kristeva (2001) makes the point that,

Klein’s depressive position offers yet another innovation, one that will eventually encourage creativity: the feeling of depression mobilizes the desire to *make reparation to* objects. The baby, by believing that he is responsible for the loss of his mother, also imagine that he can undo the nefarious effects of his aggression through her love and care for him. “The depressive conflict is a constant struggle between the infant’s destructiveness and his love and reparative impulses.” (Segal, 1990, p. 60). To deal with the depressive suffering that results from his feeling of having damaged the external and internal object, the baby tries to make reparation and restoration to the good object. His love only grows in the process. (p. 79)

The fact that the move into, or rather the overlaying over [the paranoid-schizoid position] of the depressive position, is interdependent with affective-semiotic-discursive dimensions of normativity underpins affective developmental psychoanalytic thought (Green, 1999). Keeping in mind that from the outset we are dealing with a mother-child unit, the initial precursors to any process that leads to awareness involves something that you might call an affect-laden emotional mirroring that constitutes the interactive/participative expression of the mother-infant unit (i.e., we need to remember, there are initially no separate entities). To paraphrase Winnicott’s description of the infant’s initial experience, “When I look I am seen [*by somebody else*], so I exist; I can now afford to look and see” (emphasis added). This apparently simplistic phrase requires some unraveling.

What is being suggested is that the baby sees herself and gradually attains some intuition of the “self” through the reflection seen. Initially, what you see (about your “self”) is what the mother sees:

What does the baby see when he or she looks at the mother’s face? I am suggesting that, ordinarily, what the baby sees is himself or herself. In other words, the mother is looking at the baby and *what she* (the baby) *looks like is related to what she* (the mother) *sees there*. All this is too easily taken for granted.’ (Winnicott, 1971, p. 151 [Italics in the original]).

It is important to understand that what “she sees there” – will be the mother’s own projections, wishes and desires regarding the “baby-entity.” Needless to say, these projections and desires are saturated, in fact interdependent with, the particular cultural discourses prevailing in any particular context (see Demuth et al., 2012; for interesting examples of parental discursive difference across cultural contexts). And when the baby moves from existing (through being seen), and begins to “look and see,” what the infant sees (seeing with) is already colored by the desire or intentionality of the mother - - the infants experience of the mother’s desire for it to exist [as an infant]; in other words, something akin to: “‘I want them to want me’ and that is the condition for my wanting them.” It is this complex interpenetration that forms the basis for the suggestion that the “inside” that is coming into being is already interdependently saturated with the “outside” (what is reflected back – through action, discourse, social semiosis of all forms).

In order to highlight the significance of action, transaction and affect in these mother–child dynamics Winnicott (1960) introduced the idea of a transitional space – which despite the everyday connotation that this amounts to something that exists *between* individuals, should be understood as both within-and-without – as well as potentially present (internal) even when the young child is on their own. As an example of the affective dynamics of the transitional space, Winnicott (1974) for example, proposed that through the projection out of, and onto, the *object* the child produces the conditions which allow recognition of “feeling”(s) possible. Here, the use of the phrase “produces the conditions” is significant because it is not as if the child is first feeling bad and then simply “puts the badness” outside. Rather, it is the projection that amounts to a defense against the badness (the infant or young child represses the recognition of the “internal” badness’ – caused by hunger, aggressive impulse, constitutional characteristics or whatever - by spontaneously producing the projection). And then, once projected outside, it can then (the badness) be recognized as something “not very nice” but now, and very importantly no longer “inside,” but instead controllable and containable by being “in the other,” or “in the object.”

One can begin to see the significance of the idea of the transitional space for understanding affect, emotion, the identification of feeling(s) and how such experiences are related to the emergence of self-hood. In other words, in order to know that what is being experienced is “feeling” or affect never mind identifying what that feeling is, this will involve object-relations – interacting with others and objects within a transitional space. Such an approach to subjectivity is echoed in the work of Hollway (2006) who argues that we are psycho-social “because we are products of a unique life history of anxiety- and desire- provoking life events and the manner in which they have been transformed in internal reality…(and)…because unconscious defences are an intersubjective process.” (p. 466).

It was with reference to transitional dynamics that Winnicott (1974) suggested the mother should be seen as the infant’s psychological matrix. In the beginning the mother provides the psychological or mental space within which the infant generates experience. Only gradually does the maternally provided psychological matrix begin to erode, and the infant tentatively initiates his/her own psychological matrix, one within which she/he develops the capacity to deal with separateness. What we have then is this gradual transformation from “mother-as-environment” toward “mother-as-object.” As the infant gradually attains individuation or awareness of “I-ness” she/he simultaneously begins to recognize the separateness of “infant-mother,” i.e., infant as self/object, and mother as other/object. Psychoanalysts after Winnicott draw attention to that element of taking up (attaining) a place in the “depressive position” where the mother provides “presence but absence” i.e., paradoxically being physically present with the child yet psychologically absent and contrastively, being psychologically present with/to the child and yet physically absent. Ogden (1992) highlights the ambiguous nature of this process, commenting,

This paradox can be understood in the following way: the mother is absent as object, but is there as the unnoticed, but present containing space in which the child is playing. The mother must not make her presence as object too important, for this would lead to child to become addicted to her as omnipotent object. The *development of the capacity to be alone* is a process in which the mother’s role as invisible co-author of potential space is taken over by (what is becoming) the child. In this sense, the healthy individual, when alone, is always in the presence of the self-generated environmental mother. (p. 182) [emphasis added]

The proposal that the “internal,” private experiential domain is in effect initially interpenetrated with the experiences of another (the mother) necessitates accommodating a somewhat paradoxical way of thinking – certainly one at odds with ideas on the construction of the self in cognitive-developmental psychology (e.g., Harter, 1999; Pfeifer and Peake, 2012). The suggestion that the formulating elements of a sense of self are in effect co-authored, with the infant initially possessing no recognition of what is going on, points to a certain ambiguity regarding symbolization and the social-semiotic basis of self-ness. In a way this could be understood as the sense of self forever containing the “shadow of the other” (mother) in addition to the observation that entering or taking up a self-position in discourse and language presupposes the appropriation of the available discourses in context.

## Concluding Comments

Part of the impetus for this review topic is the concern with examining the interdependence between psychological phenomena and the discursive and embodied practices of social interaction. Earlier the suggestion was made that part of the reason why CA has particular difficulties with addressing the relationship between affect and action, is the avoidance or elision of discussion regarding any construct or concept that presupposes an interiority of mental states, internal psychic life or inaccessible affective state. Such a view is certainly defensible given the suspicion over the excessive formalism of contemporary cognitive science allayed with debatable ascriptions regarding the causal dynamics of the cognition-emotion-behavior link (Coulter, 1999). It is also defensible given the participant-oriented empirical criteria of CA and the associated skepticism regarding over-interpretation. But at the same time, there may be a sense in which CA, in avoiding theoretical dialogue with psychoanalytic thought is missing an opportunity to examine implicit presuppositional assumptions that may lie behind adopting agnostic positions. In other words, the manner in which elision take place seems to result in there nevertheless remaining (if “neutral”) an implicit model of the infant/child mind, e.g., a resource identifying pattern detecting entity (Wootton, 1997; Lerner et al., 2011). The participant-oriented evidential requirements of CA may engender a reticence to consider what alternative, if somewhat marginalized perspectives such as object-relations theory and psycho-social approaches might have to offer. The proposal is that CA may find in psychoanalytic thinking a fruitful framework for understanding the interdependence between affect and action.

A number of possible directions for an empirically grounded CA theory of personhood/subjectivity could emerge from studies that for example, examine in detail the earliest moments of parent-infant engagement, documenting and explicating the multi-modal dimensions of participation, action, and affect. Mondada (2019) in a recent commentary on expanding multi-modal analysis in CA, makes that point that participants engage with their bodies not only to communicate with each other, but also in sensing the world – arguing that multisensorial practices are intersubjectively organized. Infant-focused CA studies of the earliest sensorial experiences following birth might help highlight how the status of “subjectivity” or personhood comes about in the first instance. Furthermore, examining these earliest moments longitudinally would help identify the circumstances within which the presuppositional grounding of the “infant-as-subject” begins to emerge. One could also begin to examine, given Winnicott’s (1968) argument, whether in the talk and discourse that mothers and fathers first direct toward infants, we find evidence of culturally specific representations of idealized infancy (e.g., along lines similar to Demuth et al., 2012). Finally, we might conjecture that if whatever we understand as unconscious defenses are in fact intersubjectively constituted, then by examining early parent–child interaction at a sufficiently level of granularity, we should be able to highlight those circumstances whereby the infant/young child learns what *not* to say – learning that there is much beyond language that needs to be kept under control (inappropriate actions, non-verbal misdeeds and associated behaviors that might contravene the “doing being ordinary” of everyday members’ methods).

By way of a concluding comment one can say that in the work of psychoanalysts such as Klein and Winnicott we are presented with a thoroughly relational conception of human nature. Phenomenologically, psychic life is already socially (and discursively) saturated. From this perspective it would seem that some form of affective-normativity suffuses the earliest experiences of the infant’s life, given that from the outset identificatory phenomena (introjection and projection) are not only part and parcel of embodiment, but are also conveyed, recognized and produced within and through the prevailing discourses, which constitute social action. However, this is not to presuppose the methodological shadow of a discursive or CA “master-discourse.” Hollway (2011) adopting a psycho-social perspective and commenting on the growing need to go beyond the “empty” subject of discourse analysis, argues that inner psychic processes are not “purely psychological,” if that means sealed off from the external world, and that the boundaries between the inner and outer are porous, neither autonomous or static, and that psychoanalytic theory,

specifies the way processes like splitting and identification act on social and cultural material (through meaning making and the expression of agency in practices). It does provide accounts of “how internal mental contents might be transformed” (citing Wetherell, 2003, p. 115; Hollway, 2011, p. 11).

Essentially, the proposal developed above highlights the tantalizing possibility that all “psychic” or psychological life (whatever we understand that to be) is somehow inherently social or infused with a socially shared normativity. We are of course still left with the challenge of how the affect-laden socially normativity that may underpin embodied parent-infant practices seen at the beginnings of life, finds expression – particularly given the suggestion that the essence of affect is “its capacity to seep into other domains and inhabit them” (Green, 1999, p. 285). Contemporary research in child-focused CA appears to be explicating how such seepage finds expression.

## Author Contributions

The author confirms being the sole contributor of this work and has approved it for publication.

### Conflict of Interest Statement

The author declares that the research was conducted in the absence of any commercial or financial relationships that could be construed as a potential conflict of interest.

## Footnotes

^1^The question of methodology and what counts as defensible empirical support across EM/CA, developmental psychology, and psychoanalytic research rests on the specifics of each research tradition – i.e., concerns will reflect prevailing criteria regarding appropriateness, defensibility, and correctness within each approach. Methodological observations regarding contrasts and comparisons are beyond the scope or focus of this paper.

^2^One might think of this is a place where all that is experienced is the illusion of complete control or the experience of pain (hungry – feeding happens; or hungry – pain/abandonment).

^3^This movement from one position to another should not be seen as diachronic stage-like development. Instead, it is more akin to synchronic transformation, one where the initial experiences of the paranoid-schizoid position are overlaid with the depressive position, yet remain psychologically forever recoverable. The layering of positions is a more apt metaphor compared to the idea of a stage-like transition.
